# Evaluation of Clinical and Ultrasonographic Parameters in Psoriatic Arthritis Patients Treated with Adalimumab: A Retrospective Study

**DOI:** 10.1155/2012/823854

**Published:** 2012-05-09

**Authors:** M. Teoli, A. Zangrilli, M. S. Chimenti, M. Talamonti, M. Bavetta, D. Graceffa, R. Perricone, S. Chimenti

**Affiliations:** ^1^Department of Dermatology, University of Rome “Tor Vergata”, Viale Oxford 81, 00133 Rome, Italy; ^2^Department of Rheumatology, University of Rome “Tor Vergata”, Viale Oxford 81, 00133 Rome, Italy

## Abstract

*Objectives*. The aim of this study was to evaluate clinical and US-PD parameters in PsA during adalimumab treatment. *Methods*. A retrospective study has been conducted in forty patients affected by moderate-to-severe peripheral PsA. Clinical, laboratory, and US-PD evaluations were performed at baseline, after 4, 12, and 24 weeks of treatment. They included erythrocyte sedimentation rate (ESR), C-reactive protein (CRP), visual analogue scale (VAS), Health Assessment Questionnaire (HAQ) modified for Spondyloarthritis, Psoriasis Area Severity Index (PASI) score, the 28-joint Disease Activity Score (DAS 28), and US-PD assessment. US-PD findings were scored according to a semiquantitative scale (ranging 0–3) for synovial proliferation (SP), joint effusion (SE), bone erosions (BE), and PD. *Results*. Data obtained for clinical, laboratory findings and US-PD evaluation showed statistical significant improvement in all the measures performed except for BE. A significant parallel decrease in SE, SP, and PD values were demonstrated. *Conclusion*. This study demonstrated that US-PD is a valid technique in monitoring the response to adalimumab in moderate-to-severe PsA.

## 1. Introduction

Psoriatic arthritis (PsA) is an inflammatory arthritis that occurs in up to one-third of patients with psoriasis and is usually diagnosed years after the appearance of cutaneous psoriasis [1]. Joint involvement in these patients is a potentially debilitating disease that may affect enthesis, small and large joints, and axial skeleton [2]. In particular, more than half of the patients exhibit progressive erosive arthritis, associated with severe functional impairment [3].

Despite clinical improvement with current disease modifying antirheumatic drugs (DMARDs), PsA results in radiological damage in up to 47% of patients at a median interval of 2 years [4]. Overexpression of tumor necrosis factor-*α* (TNF*α*) is believed to play a key role in the pathogenic mechanisms linking psoriasis and arthritis [5]. Similar to rheumatoid arthritis (RA), several trials in PsA have shown excellent clinical results with anti-TNF*α* agents such as etanercept [6, 7], infliximab [8], and adalimumab [9, 10].

Adalimumab is a recombinant, fully human monoclonal antibody (IgG1) that binds with high affinity and specificity to TNF-*α*. This process of neutralization of TNF-*α* by a specific monoclonal antibody should improve both skin and joint manifestations of PsA [11].

The development of new devices for assessing activity of articular involvement in PsA is a relevant topic of research in rheumatology and more recently in dermatology. Among these equipment, ultrasonography (US) is one of the most interesting tool. Iagnocco et al. demonstrated the efficacy of adalimumab treatment not only in terms of clinical and laboratory remission but also in terms of US parameters in patients with RA [12]. Moreover, a study conducted by Fiocco et al. evaluated the therapeutical response to etanercept by the US assessment of rheumatoid and psoriatic knee synovitis [13]. In fact, ultrasonography is a routinely available and noninvasive technique to evaluate bone, cartilage, tendons, ligaments, and surrounding soft tissue and relatively inexpensive bedside imaging modality with high patient acceptability. Furthermore, it allows the scanning of all peripheral joints as many times as required in monitoring early and active PsA [14].

This method allows the assessment in psoriatic joint of both inflammation-related changes such as synovitis, tenosynovitis, enthesitis, and bursitis and structural damage such as cortical bone erosions. The application of power Doppler (PD) can estimate the increase in synovium, tendon sheaths, bursae, and enthesis perfusion due to a disorganized pattern of blood vessels formation that is typical of PsA [15].

The aim of this study was to evaluate the use of US and PD (US-PD) technique in monitoring joint involvement in moderate-to-severe PsA patients treated with adalimumab and to confirm the presence of an association between US and clinical and serological findings.

## 2. Materials and Methods

From January 2008 to December 2009, a retrospective study has been conducted in our collaborative outpatients unit of Dermatology and Rheumatology at the University of Rome “Tor Vergata.” Forty patients (21 males and 19 females), aged between 33 and 76 years, affected by moderate-to-severe peripheral PsA with cutaneous manifestation of psoriasis were studied. Clinical diagnosis was made using CASPAR (ClASsification criteria for Psoriatic ARthritis) criteria for PsA [14]. Patients underwent treatment with adalimumab if they were unresponsive to or had contraindications for at least two other conventional systemic treatments (methotrexate, ciclosporin, leflunomide, sulphasalazine). All patients were screened by chest radiography, laboratory tests (including screening for hepatitis A, B, and C viruses), and PPD skin test. Prior to treatment, all patients signed written informed consent. Adalimumab was administrated with a dose of 40 mg subcutaneously, every other week. Clinical, laboratory, and US-PD evaluations were performed at baseline (T0), after 4 (T4), 12 (T12), and 24 (T24) weeks of treatment. They included a general physical examination, erythrocyte sedimentation rate (ESR), and C-reactive protein (CRP) with normal range of 0–15 mm/h and 0–0.5 mg/dL, respectively. Disease activity assessment was made using visual analogue scale (VAS), Health Assessment Questionnaire modified for Spondyloarthritis (SpA-HAQ), Psoriasis Area Severity Index score (PASI), the 28-joint Disease Activity Score (DAS28-ESR), and US-PD.

Patients' demographics and clinicolaboratory characteristics as well as previously performed treatments are summarized in Table 1.

At baseline, patients' evaluations were performed by a dermatologist and a rheumatologist for both cutaneous and joint disease.

The joint considered by the physician as the most involved by arthritis was evaluated. The selected joint, the same for each patient, was scanned by US-PD: 10 metatarsophalangeal (3 II finger and 7 toe right and left), 18 metacarpophalangeal (8 II finger, 7 III finger, and 3 IV finger right and left), 8 distal interphalangeal (5 II finger and 3 III finger right and left), and 4 proximal interphalangeal (1 II finger, 2 III and IV finger, and 1 V finger right and left).

### 2.1. US-PD Evaluation

US was performed with Logiq 5 Pro (GE Healthcare, Milwaukee, USA) using a high-frequency —12 MHz—linear array transducer with power Doppler unit. The US study was performed by an experienced rheumatologist sonographer (X) who was blinded to the clinical and laboratory findings in each patient. Each joint was examined by performing both longitudinal and transverse scans. In addition, power Doppler was used with the following settings: PRF 0.7 KHz gain 18–30 dB, low filter. The color box was restricted to the vascular area studied. Intra-articular color density was quantified in the longitudinal and transverse views within a user-defined region of interest (ROI). Using a multiplanar scanning technique, the presence of any inflammation-related change in the joints and/or in the adjacent tendon sheaths and bursae synovial effusion (SE), synovial proliferation (SP), increased local perfusion by power Doppler) was assessed. The presence of permanent joint damage bone erosions (BE) was also registered.

All the changes within each articular and periarticular structure both on B-mode and PD US were recorded as being present in accordance with the OMERACT definitions of Ultrasound Pathology.

PD signal intensity was graded with a scale (ranging 0–3): grade 0: no signal; grade 1: less 1/3 of ROI; grade 2: less 2/3 of ROI; grade 3: more 2/3 of ROI on a zone of synovial hypertrophy.

Furthermore, for all the changes in B-mode, a semiquantitative score (0–3) was used for each structure examined indicating the degree of inflammatory activity and structural damage (0: normal; 1: mild change; 2: moderate change; 3: severe change). For the BE score we consider the following values: grade 0: absence of erosions; grade 1: 1-2 erosions; grade 2: >2 erosions; grade 3: large destructed area. US-PD measures are summarized in Table 2.

### 2.2. Statistical Analysis

Data from the clinicolaboratory analyses were entered into a Windows-based database (Microsoft Excel 2007) and all statistical analyses were performed using the statistical software GraphPad Prism 5 statistical software (GraphPad Software, San Diego, CA). Data were expressed as mean ± standard deviation (SD). The significance of differences in mean values obtained at T0, T4, T12, and T24 weeks of treatment was assessed with Student's *t*-test (statistical significance was set at *P* ≤ 0.05).

## 3. Results and Discussion

### 3.1. Patient Characteristics

Forty patients (21 males and 19 females) with moderate-to-severe PsA were recruited in the study. The mean age was 51.5 years (mean ± SD: 51.5 ± 10.3, range 33–76), and the duration of the disease ranged from 1 to 31 years (mean ± SD: 10.7 ± 7.7).

With respect to baseline findings, at all follow-up examinations (weeks 4, 12, and 24), a significant reduction in all clinical, laboratory, and ultrasonographic (*P* ≤ 0.005) parameters of disease activity was found.

Findings on the clinical, laboratory, and US-PD parameters assessed throughout the study are shown in Table 2.

### 3.2. Clinical Findings

The disease activity, as expressed by the DAS28-ESR, revealed a decrease from baseline (mean DAS28-ESR of 4.6) to T4 (mean DAS28-ESR of 4.3) and a statistical significant reduction at T12 (mean DAS28-ESR of 3.7) and T24 (mean DAS28-ESR of 3.6) was observed.

The PASI score showed at baseline a mean value of 9.2. We observed a significant improvement in mean PASI score: from 4.6 at T4 to 1.8 at T12 and maintains 1.8 at T24.

Patient's clinical assessment for pain showed a statistical significant reduction in mean pain VAS values from 65.0 at T0, 50.4 at T4, 34.4 at T12, to 12.3 at T24.

The mean SpA-HAQ functional disability index revealed a dramatical impairment from 1.1 at baseline to 0.5 at 4 weeks and from 0.1 at 12 weeks to 0.0 at 24 weeks.

No patients required additional drugs during the 24 weeks of followup.

The safety profile of the drug during the treatment was satisfactory. The most frequently complained adverse event that was an injection site reaction observed in 7 patients during treatment. Five patients referred a urinary tract infection was resolved with an antimicrobial oral treatment and did not require the discontinuation of adalimumab.

### 3.3. Laboratory Findings

The mean ESR decreased slowly from baseline (value of 34.7) to T4 (value of 29.3); instead we observed a significant reduction of mean ESR from 20.7 at T12 to 6.8 at T24.

Likewise, the mean CRP values showed a specific trend with just a partial reduction from 20.8 at T0 to 18.9 at T4. At T12 and at T24 we observed a statistical significant decrease in mean CRP values that were, respectively, 13.1 and 5.1 (*P* ≤ 0.005).

### 3.4. US Findings

According to the data analysed for clinical and laboratory findings, our US-PD evaluation showed a statistical significant improvement in all the measures performed except for BE.

We observed that the most involved joints examined demonstrate signs of inflammation at baseline: mean SE was 2.3, mean SP was 1.12, and mean PD score was of 2.5 in all the 40 of 40 joints (100%).

A statistical significant improvement of mean values of SE, SP, and PD was achieved. At T4 mean SE was 1.5, mean SP was 0.9, and mean PD was 1.8. At T12 mean SE was 0.4, mean SP was 0.15, and mean PD was 1.1. At T24 mean SE was 0.1, mean SP was 0.025, and PD was 1.0.

**Figure 1:**
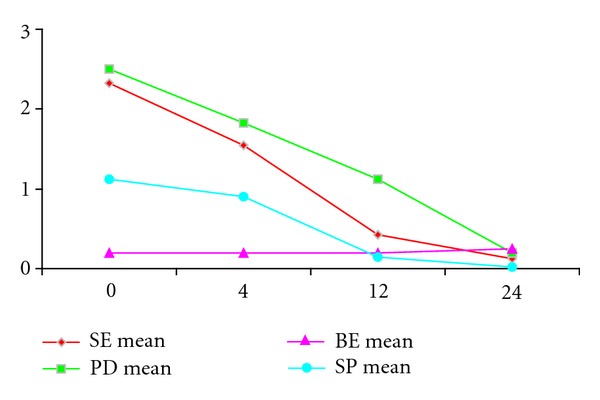
US-PD evaluation.

A significant parallel decrease in SE, SP, and PD values was demonstrated. On the contrary, no modifications on BE measures were observed during the study as appeared in Figure 1.

**Figure 2:**
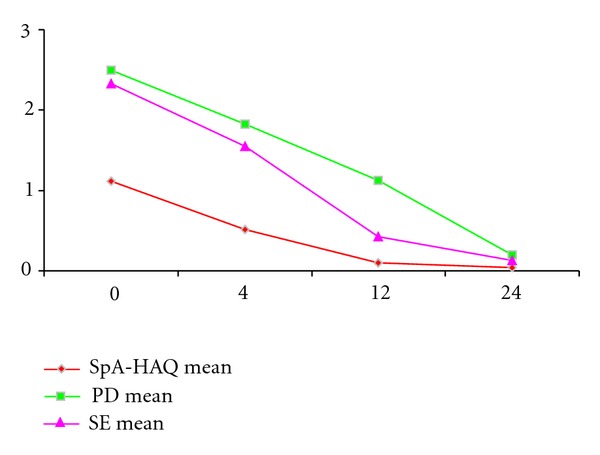
Parallel decrease in US, PD, and SpA-HAQ parameters at week 12.

A significant parallel decrease in US, PD, and SpA-HAQ parameters was found at the assessment at week 12 (*P* < 0.0005 for within-subject between-visit changes in each parameter) as shown in Figure 2.

**Figure 3:**
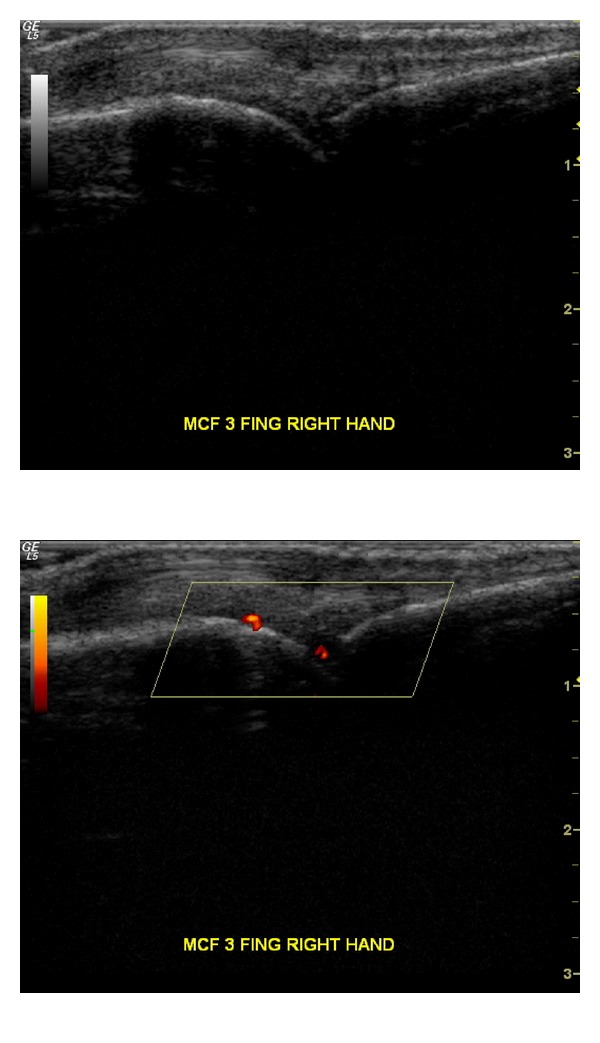
MCF joint with synovial effusion grade 2 and cPD signal grade 3 at baseline.

**Figure 4:**
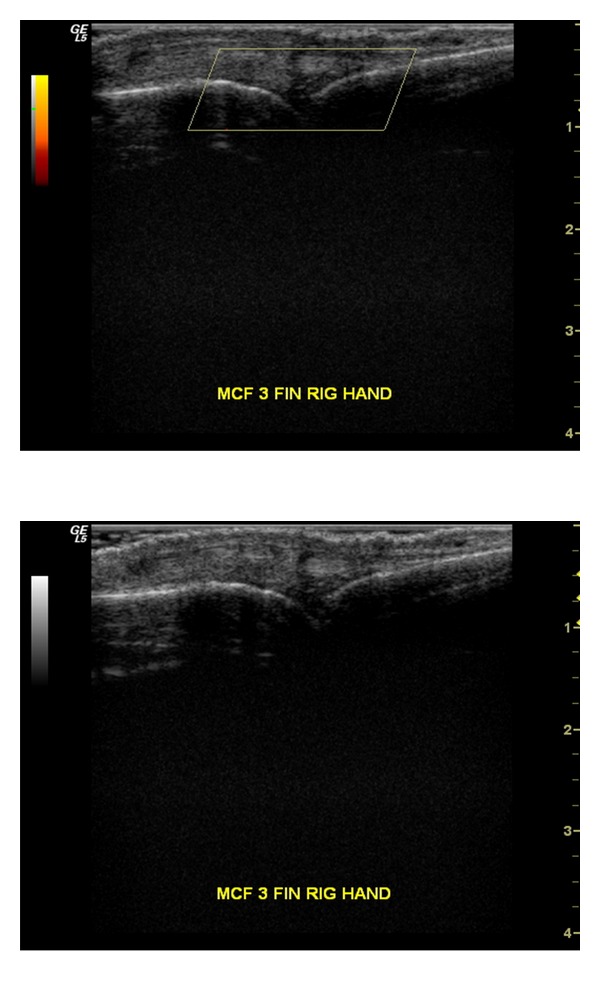
MCF joint with synovial hypertrophy grade 0 and cPD signal grade 0 at week 24.

Representative images of US and PD changes in an MCF joint after adalimumab treatment are shown in Figures 3 and 4.

## 4. Discussion

The development of anti-TNF*α* therapies, which target a specific cytokine of the immune system, has dramatically changed the treatment of psoriasis and psoriatic arthritis over the last years. Tumor necrosis factor (TNF*α*) blockers have been verified to be useful in the management of many clinical disease expressions of PsA including peripheral arthropathy, axial involvement, enthesitis, and cutaneous manifestations [16, 17]. Considering the limited data regarding the efficacy of conventional therapies in preventing radiographic progression of PsA and in ameliorating the disability in PsA patients, adalimumab therapy represents a significant advance in the treatment of both skin and joints. In fact, adalimumab monotherapy, at a dosage of 40 mg subcutaneously every other week, is an effective and safe treatment with a rapid onset of action for the management of PsA, as confirmed the data reported before [18]. As demonstrated in several studies, US and PD are able to detect synovial vascularization and can be considered a routinely available, noninvasive, and relatively inexpensive bedside imaging modality with high patient acceptability, which allows the scanning of all peripheral joints as many times as required in inflammatory joint disease [19]. Recently, the use of power Doppler and the technological improvement of US equipment have further shown the usefulness of this diagnostic tool in the evaluation of arthritis [12, 20, 21].

Gutierrez et al. reported the validity, responsiveness, and predictive value of power Doppler ultrasonography monitoring of response to TNF*α* blockers in a long-multicentric study of 367 RA patients. Moreover, the persistence of synovial PD signal appears to have predictive value in relation to radiological progression in patients with established RA who are treated with anti-TNF*α* therapy [22].

In a longitudinal study of 20 patients made by Fiocco et al., the efficacy of etanercept in rheumatoid and psoriatic knee joint synovitis (KJS) was determined by assessing the time-dependent changes in disease activity and in combined grey scale and power Doppler ultrasonographic outcome measures [13].

Our study represents the first attempt to investigate, by clinical, laboratory, and US-PD evaluation, the effects of adalimumab therapy in moderate-to-severe PsA patients. Adalimumab efficacy was consistent as expressed by the improvement of the disease activity parameters, and the safety profile was excellent. The changes in clinical and ultrasonographic findings were concordant in showing a sustained reduction in disease activity indices, as DAS28-ESR, PASI, and SpA-HAQ particularly at week 12. At this time, the reduction in ultrasonographic measures (SP, SE, and PD) appeared highly significant as shown in Figure 3.

Our study suggests that US-PD is a useful and sensitive imaging instrument for assessing the response to treatment with adalimumab of synovitis. We have reported that the most involved joint is suitable to be assessed by US-PD for detecting improvements in SP and in SE induced by the treatment. On the contrary, no changes in BE were observed, probably for the conciseness of the study.

Actually, no clearly predictive makers of response to biological agents are established as well as there are no specific tools to define the remission of PsA during treatment. In this regards, US-PD changes have been shown to be complementary to the standard clinical evaluation in joints assessment and to provide similar profiles of systemic disease activity indices in monitoring response to treatment.

## 5. Conclusion

Our experience, although preliminary, demonstrated in a consistent number of PsA patients that US-PD is a valid technique in monitoring the response to adalimumab in moderate-to-severe PsA. Indeed, this diagnostic method has many advantages over other procedures to analyze clinical indices of disease activity [23]. We reported a link between US-PD findings and the frequently used clinical and serological indices of disease activity. This confirmed the use of US-PD as one of the options in monitoring clinical efficacy of anti-TNF*α* therapy in PsA [24, 25]. To the best of our knowledge, there are no reports of sustained regression of ultrasonographic synovial involvement after adalimumab treatment in moderate-to-severe PsA resistant to other synthetic DMARDs treatment. Further studies are needed to better evaluate the role of US-PD in monitoring the efficacy of anti-TNF*α* treatment in PsA and to compare the efficacy between the other anti-TNF*α* blockers.

## Figures and Tables

**Table 1:** Patients demographics and disease characteristics at baseline.

Number of patients	40
Male/female	21/19
Age, (mean ± SD)	51.5 ± 10.3
*Duration of disease*	
Psoriasis (mean ± SD)	22.2 ± 11.5
Psoriatic arthritis (mean ± SD)	10.7 ± 7.7
PASI (mean ± SD)	9.2 ± 10.8
Pain VAS (mean ± SD)	65 ± 21.3
DAS 28 (mean ± SD)	4.6 ± 1.5
ESR (mean ± SD)	34.7 ± 21.6
CRP (mean ± SD)	20.8 ± 16.4
*Age of onset (mean ± SD)*	
Psoriasis (mean ± SD)	29.3 ± 16.5
Psoriatic arthritis (mean ± SD)	40.8 ± 12.2
Previous methotrexate treatment	31 (77.5%)
Previous ciclosporin treatment	35 (87.5%)
Previous pUVA-UVB treatment	14 (35%)
Previous leflunomide treatment	17 (42.5%)
Previous sulphasalazine treatment	12 (30%)

**Table 2:** Clinicolaboratory parameters and US-cPD measures.

	Baseline	T4	T12	T24
ESR mean (range)	34.7 (6–79)	29.3 (5–68)	20.7 (1–76)	6.8 (2–17)
CRP mean (range)	20,8 (1.6–68.9)	18,9 (1.1–64.7)	13,1 (1.1–48.3)	5,1 (0.2–23.9)
Pain VAS mean (range)	65,0 (13–100)	50,4 (0–98)	34,4 (0–87)	12,35 (0–72)
SpA-HAQ mean (range)	1.1 (0.8–2.3)	0.5 (0.1–1)	0.1 (0–0.8)	0.0 (0–0.3)
PASI mean (range)	9.2 (0–61.8)	4.6 (0–34.5)	1.8 (0–21.2)	1.9 (0–13.9)
DAS 28-ESR mean (range)	4.6 (2.8–7.7)	4.3 (2.6–6.9)	3.7 (2.1–5.8)	2,6 (1.4–3.8)
SP mean (range)	1,125 (0–2)	0,9 (0–2)	0,15 (0-1)	0,025 (0-1)
SE mean (range)	2.3 (1–3)	1.5 (0–3)	0.4 (0–2)	0.1 (0-1)
BE mean (range)	0,2 (0–2)	0,2 (0–2)	0,2 (0–2)	0,25 (0–2)
cPD mean (range)	2.5 (1–3)	1.8 (0–3)	1.1 (0–3)	1 (0–2)

## Abbreviations

PsA: Psoriatic arthritis
RA: Rheumatoid Arthritis
TNF*α*: tumor necrosis factor-*α*
DMARDs: disease modifying anti-rheumatic drugs
CASPAR criteria: (ClASsification criteria for Psoriatic ARthritis)
ESR: Erythrocyte sedimentation rate
CRP: C-reative protein
VAS: Visual Analogue Scale
SpA-HAQ: Health Assessment Questionnaire modified for Spondyloarthritis
PASI: Psoriasis Area and Severity Index
DAS28-ESR: 28 joint Disease Activity Score
US: Ultrasonography
PD: power Doppler
ROI: region of interest
SP: synovial proliferation
SE: synovial effusion
BE: bone erosions
SD: standard deviation.
